# Effect of Wuzi Yanzong on Reproductive Hormones and TGF-β1/Smads Signal Pathway in Rats with Oligoasthenozoospermia

**DOI:** 10.1155/2019/7628125

**Published:** 2019-04-16

**Authors:** Zhuo Yang, Xi Zhang, Zhimin Chen, Changjiang Hu

**Affiliations:** ^1^Medicine Institute, Chengdu University of Traditional Chinese Medicine, Chengdu 611137, China; ^2^Department of Pharmacy, Chengdu Women's & Children's Central Hospital, Chengdu 610074, China; ^3^Chengdu Institution of Chinese Herbal Medicine, Chengdu 610016, China

## Abstract

**Introduction:**

Wuzi Yanzong (WZYZ) formula, a famous traditional Chinese medicinal prescription, has been widely used to treat kidney essence insufficiency-induced oligoasthenozoospermia in ancient and modern clinical practice. Previous studies have demonstrated that WZYZ formula exhibits significantly therapeutic activity. The aim of this study was to evaluate the effect of WZYZ formula on the reproductive hormone levels and the TGF-*β*1/Smads signal pathway in the testis, to explore the underlying mechanisms of WZYZ formula to improve spermatogenic function of testis in rats with oligoasthenozoospermia.

**Materials and Methods:**

In order to control the quality of the drug, the main components of the WZYZ formula were analyzed by HPLC. A rat model of oligoasthenozoospermia was established, by daily administration of tripterygium glucosides for 4 weeks, and treated with 1.62g/kg of WZYZ formula. The testes were histopathologically examined and serum levels of gonadotropin release hormone (GnRH), estradiol (E2), follicle-stimulating hormone (FSH), testosterone (T), and luteinizing hormone (LH) were measured by ELISA. TGf-*β*1, Smad2, and Smad4 mRNA and protein levels in the testis were evaluated by immunohistochemistry (IHC), quantitative real-time RT-PCR (qRT-PCR), and Western blotting (WB).

**Result:**

Oral administration of WZYZ formula restored testicular structure and significantly increased the histology score in the oligoasthenozoospermic rats. In addition, WZYZ also significantly increased the serum levels of GnRH, LH, E2, and T and decreased that of FSH. Meanwhile, TGf-*β*1, Smad2, and Sma4 expression levels were significantly decreased.

**Conclusions:**

WZYZ alleviates oligoasthenozoospermia by restoring the reproductive hormones and targeting the TGf-*β*1/Smads pathway.

## 1. Introduction

Lifestyle changes, environmental pollution, and psychological factors have significantly increased infertility rates in the general population to 10-15%, with male infertility accounting for 50% of the cases [1, 2]. In addition, the incidence of male infertility is continually increasing year after year, resulting in significant challenges for many couples wishing to conceive. Studies show that the main cause of male infertility is oligoasthenozoospermia, which is defined by the World Health Organization (WHO) as fewer than 15×10^6^ spermatozoa per milliliter of sperm and less than 32% progressive motile (PR) or fast sperms (i.e., low sperm motility) [3]. Since the exact pathogenesis of this condition is not clear, the current therapy relies on hormones, antioxidants, zinc preparations, etc., which have resulted in poor outcomes [4–7]. In some Asian countries, traditional Chinese medicine (TCM) is a beneficial supplement to modern medicine. In recent years, TCM has been gradually recognized in many western countries. TCM also plays an important part in drug development, providing abundant novel compound lead sources. As recorded in the Su wen (basic questions) treatise on traditional Chinese medicine (TCM), the kidneys store essence, govern reproduction, and are the origin of congenital constitution. Sperm production therefore depends on the nourishment of kidney essence, which influences reproductive function, growth, and development. Accordingly, the deficiency of kidney essence is considered the main cause of oligoasthenospermia in TCM, and the treatments aim to restore and strengthen this essence. The treatment is effective and has no apparent adverse reaction.

A classical TCM formula for this type of infertility, Wuzi Yanzong (WZYZ) formula, was first recorded in the “she sheng zong miao fang” (摄生众妙方) text and is a mixture of* Fructus lycii, Semen cuscutae *(fried),* Fructus rubi*,* Fructus schisandrae* (steamed), and* Semen plantaginis *(stir fried with salt) seeds in the ratio of 8:8:4:1:2. It nourishes the kidney and strengthens the essence and therefore has been used to treat oligoasthenozoospermia secondary to kidney essence insufficiency as “The Number One Herbal Formula for Assisting Fertility” [8]. Modern pharmacological studies show that the WZYZ formula regulates sex hormone levels, increases sperm quantity, motility, and viability, restores sexual organ development, and inhibits testicular apoptosis [9–15]. Furthermore, clinical studies show that the WZYZ formula can significantly improve spermatogenic function, semen quality, and male fertility [16–22]. Nevertheless, the mechanisms underlying the effects of WZYZ are still unclear and need to be elucidated for incorporating it in clinical practice.

In an earlier study, we found that WZYZ formula improved the sperm motility, viability, and density in a rat model of oligoasthenozoospermia induced by* tripterygium wilfordii *(GTW), in addition to increasing the weight of the testis and seminal vesicle gland. The aim of the present study was to determine the regulatory effects of WZYZ formula on reproductive hormones and TGF-*β*1/Smads pathway in the above animal model, in order to elucidate its molecular mechanism of action.

## 2. Materials and Methods

### 2.1. Plant Materials

*Fructus lycii, Semen cuscutae*,* Fructus rubi*,* Fructus schisandrae,* and* Semen plantaginis* samples were provided by Sichuan Neo-Green Pharmaceutical Technology Development Co., Ltd. (Chengdu, China) and were identified by Professor Xianming Lu (Chengdu University of Traditional Chinese Medicine, Chengdu, China).

### 2.2. Chemicals and Reagents

The reference standards of hyperoside, quercetin, kaempferol, schisandrin, and chlorogenic acid were obtained from the National Institutes for Food and Drug Control (Beijing, China). Acetonitrile and methanol (Fisher, USA) were of HPLC-grade, and other chemicals were of analytical reagent grade. Water was distilled using a Milli-Q water purification system (Millipore, Bedford, MA). GTW tablets (10 mg per tablet) were purchased from Hunan Qianjin Synergy Pharmaceutical Co., Ltd. (Hunan, China) and methyltestosterone tablets (5 mg per tablet) from Tianjin Lisheng Pharmaceutical Co., Ltd. (Tianjin, China).

### 2.3. Preparation of the WZYZ Formula Sample

The WZYZ formula sample was prepared in our laboratory. The herbs were weighed, powdered, and passed through a 65 mesh sieve. The processed herbs were then mixed in the ratio of 8:8:4:1:2, as outlined in the Chinese Pharmacopoeia 2015 version [23].

### 2.4. Animals

Thirty-two healthy male Sprague-Dawley rats, aged 6-7 weeks and weighting 200-220g, were provided by Chengdu Dasuo Experimental Animal Co., Ltd. (Certificate: SCXK(Chuan)2015-030). All animals were housed under controlled temperature (25±2°C) and a 12h light-dark cycle and fed standard diet and water. The animal experiments were reviewed by the Committee of Scientific Research and the Committee of Animal Care of the Chengdu University of Traditional Chinese Medicine.

### 2.5. HPLC Analysis of WZYZ Sample

Chromatographic separation was performed to control the quality of the sample. The main components in the sample were quantified on a Shimadzu LC-2040C HPLC system (Shimadzu, Japan) using an Agilent 5 TC-C18 column (length 250mm, diameter 4.6 mm and particle size 5*μ*m). WZYZ liquor was prepared by dissolving 2g of the powder in 50ml of 70% methanol and sonicating for 60 min and then passed through a 0.45*μ*m filter. The HPLC separation conditions were as follows: follow velocity, 1 ml/min, injected volume, 5 *μ*l, column temperature, 30°C, and UV wave length, 250 nm. The mobile phases A and B, respectively, consisted of 10:1 acetonitrile and methanol and 0.04% phosphoric acid aqueous. The gradient elution was as follows: 0-5 min, 5-15% A; 5-15 min, 15-19% A; 15-25 min, 19-21% A; 25-35 min, 21-26% A; 35-70 min, 26-90% A.

### 2.6. Establishment of Oligoasthenozoospermia Model and Treatment

The rats were randomly divided into four groups: the untreated control, untreated model, WZYZ-treated, and positive control group. Oligoasthenozoospermia was induced for four weeks in all but the untreated control group with daily oral doses of 20 mg/kg GTW [24]. Meanwhile, WZYZ-treated and positive control groups were, respectively, given daily oral doses of 1.62 g/kg WZYZ and 25 mg/kg methyltestosterone solution (in distilled water), while the control rats and model rats received the same volume of distilled water.

The WZYZ dose for rat was calculated from the adult daily dose recommended in the Chinese Pharmacopoeia 2015 version [23]. The different treatment groups are summarized in Table 1.

After the treatment duration, the rats were sacrificed by cervical dislocation. Blood was collected from the abdominal aorta, left undisturbed for 30min and centrifuged at 3500rpm for 15 min to separate the serum. The testes were extracted and used to perform histopathology, immunohistochemistry (IHC), western blotting (WB), and quantitative real-time PCR (qRT-PCR).

### 2.7. Enzyme-Linked Immunosorbent Assay (ELISA)

The serum levels gonadotropin-releasing hormone (GnRH), follicle-stimulating hormone (FSH), luteinizing hormone (LH), testosterone (T), and estradiol (E2) were measured using specific ELISA kits (Huamei Biological Co., Ltd., Wuhan, China). The sensitivity of the ELISA kits was 1 pg/ml (GnRH), 0.07 Miu/ml (FSH), 0.15 mIU/ml (LH), 13 pg/ml (T), and 1.9pg/ml (E2). Eight samples were analyzed from each group.

### 2.8. Histopathologic Examination

The testes were fixed overnight in 4% paraformaldehyde (PFA) and embedded in paraffin. The paraffin-embedded tissues were sliced into 3*μ*m sections and stained with hematoxylin and eosin Y (HE) as per standard protocols. The histopathological alternations were graded using Johnsen's scoring [25]. Six samples were analyzed from each group.

### 2.9. Quantitative Real-Time PCR

The testes (6 per group) were flash frozen in liquid nitrogen mechanically homogenized and stored at -80°C. Total RNA was extracted using TRIZOL reagent (Thermo Fish Scientific, USA) according to the manufacturer's instructions and reverse transcribed into cDNA using the PrimeScript RT reagent Kit (Baocheng Biological Engineering Co., Ltd., China). The qRT-PCR reactions mix consisted of 10*μ*l SYBR Premix Ex Taq II, 0.8*μ*l each of the forward and reverse primers, 2*μ*l cDNA, and 6.4*μ*l ddH_2_O. The cycling conditions were as follows: 95°C for 30s followed by 40 cycles of 95°C for 5s, 55°C for 30s, and 72°C for 30s. A melting curve analysis was performed to ensure the specificity of the amplified products, and the relative gene expression was calculated using the 2^−ΔΔCT^ method with *β*-actin as the internal control. The primer sequences are shown in Table 2.

### 2.10. Immunoblotting

Proteins expression levels were detected by Western blot. Protein was extracted from the testes (6 per group) using a protein extraction kit (Beyotime Biotechnology, Shanghai, China) and quantified with the bicinchoninic acid (BCA) method (Thermo Fisher Scientific, USA). Equal amounts of protein per sample were mixed with the loading buffer, boiled for 15 min at 95°C, and loaded into a 10% sodium dodecyl sulfate polyacrylamide gel. After resolving the proteins by SDS-PAGE, the bands were transferred to PVDF membranes. The latter were blocked for 2h with 5% TBST buffer in BSA at room temperature and incubated overnight with primary antibodies against Smad2 (ab40855, 1:5000), Smad4 (ab40759,1:2000), TGF-*β*1 (ab92486,1:500), and *β*-actin (ab8226, 1:5000). The blots were washed and incubated with goat anti-rabbit IgG H&L (ab6721, 1:5000) and goat anti-mouse IgG H&L (ab6789, 1:5000) secondary antibodies. All antibodies were purchased from Abcam, UK. The immunoblots were visualized using ECL (Thermo Fish Scientific, USA) according to the manufacturer's instructions and quantified using Image Lab software.

### 2.11. Immunohistochemistry

Tissue sections were dewaxed through an ethanol gradient and incubated with 3% H_2_O_2_ in methanol for 10 min at room temperature to quench endogenous peroxidases. The slides were then boiled in 0.01M sodium citrate buffer (pH 6) for antigen retrieval, washed with PBS, and blocked with 10% normal goat serum (Beijing Zhongshan Jinqiao biological Co., Ltd., China) for 20 min at 37°C. The processed sections were incubated overnight with primary antibodies against Smad2 (ab40855, 1:40), Smad4 (ab40759, 1:40), and TGF-*β*1 (ab92486, 1:100) at 4°C followed by a 30 min incubation with the secondary antibody (Beijing Zhongshan Jinqiao Biological Co., Ltd., SP-9001, China) at 37°C. After immersing in DAB (Beijing Zhongshan Jinqiao Biological Co., Ltd., China) for color development, the sections were rinsed with distilled water, counterstained with hematoxylin, dehydrated, and mounted. The stained sections were observed under a light microscope (BA200Digital, Motic, China) at 400× magnification. Three slices per sample and three fields per slice were analyzed. The integrated optical density (IOD) was quantified using Image-pro Plus 6.0 software and the mean density (MD) of each sample was calculated. Six samples were analyzed from each group.

### 2.12. Statistical Analysis

Statistical analyses were performed using SPSS 19.0 software. Data are expressed as mean ± standard deviation (SD). One-way analysis of variance (ANOVA) was used to compare multiple groups, followed by LSD's post hoc test. The Kruskal—Wallis test was used for nonparametric data. P values < 0.05 were considered statistically significant.

## 3. Results

### 3.1. Identification of Components from WZYZ Formula

**Figure 1:**
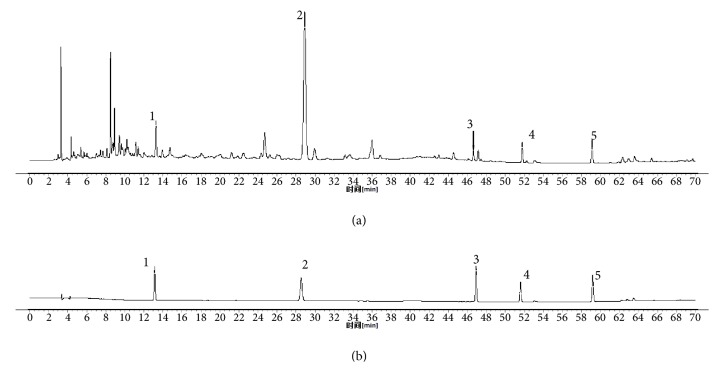
Chemical profile of WZYZ (a) and mixture of standard compounds (b) obtained by HPLC; (1) chlorogenic acid; (2) hyperoside; (3) quercetin; (4) kaempferol; (5) schisandrin.

By comparing the retention time in HPLC analysis with an accepted standard, 5 known components were identified in the WZYZ formulation (Figure 1). The contents of (2) hyperoside and (5) schisandrin were 1.889 mg and 0.46 mg, respectively.

### 3.2. WZYZ Restores the Hormonal Imbalance Induced by Oligoasthenozoospermia

**Figure 2:**
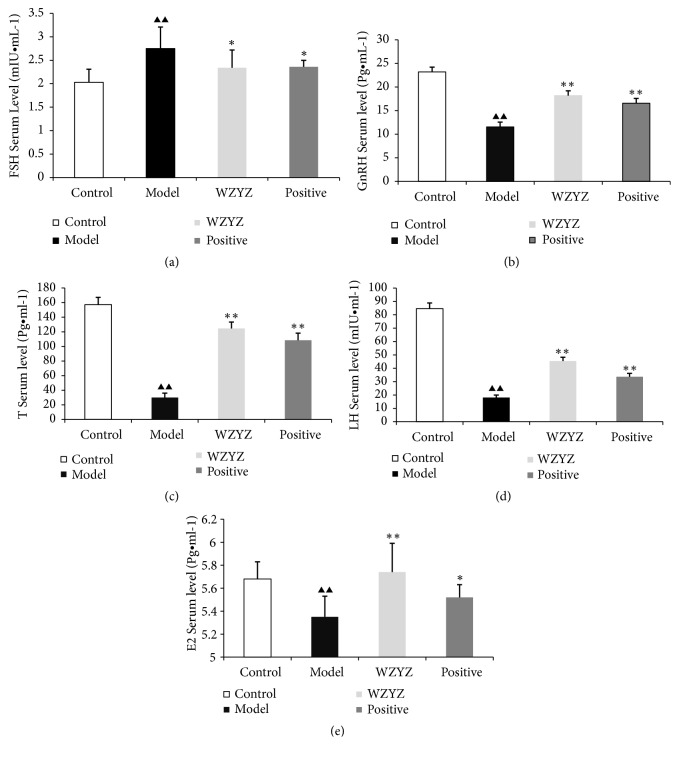
Effect of WZYZ on serum FSH (a), GnRH (b), T (c), LH (d), and E2 (e) levels. Bars represent means± SD of 8 rats per group. ^▲▲^p<0.01 versus the control group, ^*∗*^p <0.05 versus the model group, and ^*∗∗*^p <0.01 versus the model group.

As shown in Figure 2, the serum FSH (Figure 2(a)), GnRH (Figure 2(b)), T (Figure 2(c)), LH (Figure 2(d)), and E2 (Figure 2(e)) levels were significantly altered in the model group compared to the other groups (p<0.01). Oligoasthenozoospermia significantly increased serum FSH levels and decreased that of GnRH, T, LH, and E2 relative to the control group, which were restored by WZYZ formula (p<0.01) as well as methyltestosterone (p<0.05 for FSH; p<0.01 for other hormones) treatment. Thus, WZYZ restored the sex hormones to normal levels in the infertile animals.

### 3.3. WZYZ Restored the Testicular Structure in the Infertile Rats

**Figure 3:**
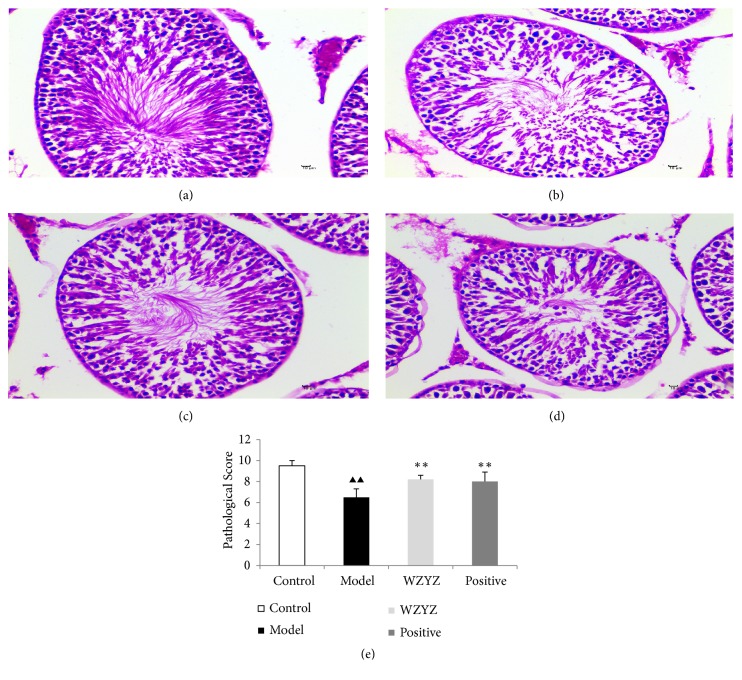
HE staining of the testis. (a) Control group: no histopathological changes. (b) Model group: spermatogenic cells arranged in disorder, reduced significantly in number. (c) WZYZ group: the number of spermatogenic cells decreased slightly. (d) Positive group: the number of spermatogenic cells decreased. (e) Histological score. Bars represent means± SD of 6 rats per group. ^▲▲^p<0.01 versus the control group; ^*∗∗*^p <0.01 versus the model group.

Significant histopathological changes were seen in the testicular tissues after 4 weeks of modelling relative to healthy tissues (p<0.01). The base membrane of the seminiferous tubule became thin, and the spermatogenic cells in the seminiferous tubules were fewer and disorganized compared to that in the healthy testes. In addition, Johnsen's scores were significantly lower in the model group relative to the control group (p<0.01). WZYZ formula significantly alleviated the tissue damage and increased Johnsen's scores in the modelled rats (p<0.01), with effects similar to that of methyltestosterone (Figure 3).

### 3.4. TGf-*β*1, Smad2, and Smad4 mRNA Expression

**Figure 4:**
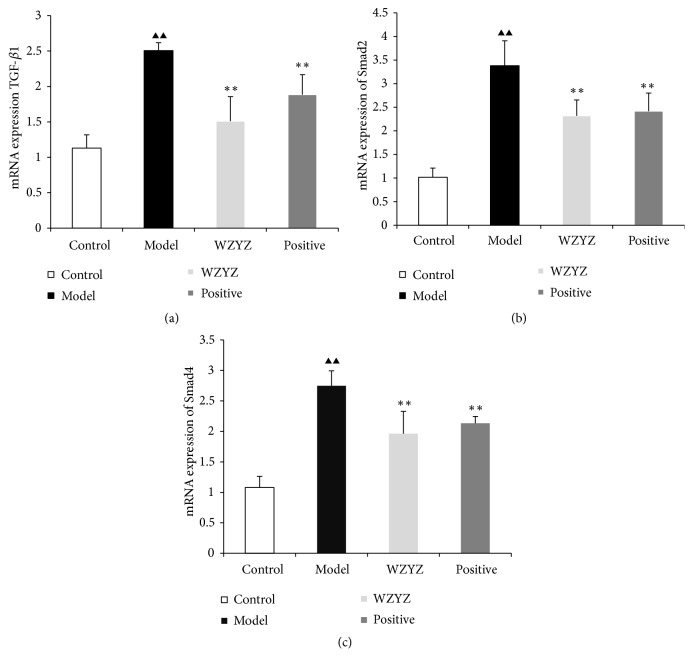
TGf-*β*1 (a), Smad2 (b), and Smad4 (c) mRNA expression in testis. Bars represent means± SD of 6 rats per group. ^▲▲^p<0.01 versus the control group; ^*∗∗*^p <0.01 versus the model group.

Next, qRT-PCR analysis was performed to assess TGf-*β*1, Smad2, and Smad4 mRNA expression in rat testis with oligoasthenozoospermia. As illustrated in Figure 4, the TGf-*β*1 (Figure 4(a)), Smad2 (Figure 4(b)), and Smad4 (Figure 4(c)) mRNA expression levels were markedly increased in the model group (p<0.01, p<0.01, and p<0.01) relative to those in the control group. Meanwhile, the TGf-*β*1, Smad2, and Smad4 mRNA expression levels were significantly downregulated in the WZYZ formula group and positive group relative to those in the model group (P<0.01, P<0.01, and P<0.01).

### 3.5. TGf-*β*1, Smad2, and Smad4 Protein Expression

**Figure 5:**
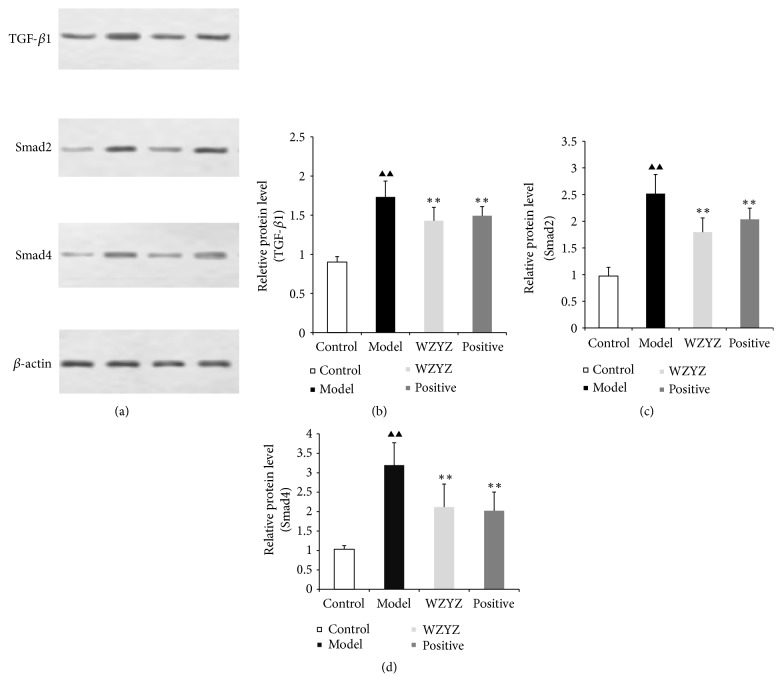
TGf-*β*1, Smad2, and Smad4 protein expression in testis. (a) Western blot of TGf-*β*1, Smad2, and Smad4 in the testicles; quantification of TGf-*β*1 (b), Smad2 (c), and Smad4 (d). Bars represent means± SD of 6 rats per group. ^▲▲^p<0.01 versus the control group; ^*∗∗*^p <0.01 versus the model group.

The effect on TGf-*β*1, Smad2, and Smad4 protein expression in testis of the rats with oligoasthenozoospermia is shown in Figure 5(a). TGf-*β*1 (Figure 5(b)), Smad2 (Figure 5(c)), and Smad4 (Figure 5(d)) protein expression increased markedly in the model group relative to those in the control group (p<0.01, p<0.01, and p<0.01). The TGf-*β*1, Smad2, and Smad4 protein expression were significantly downregulated in WZYZ formula group (p<0.01) and methyltestosterone group relative to those in the model group (p<0.01).

### 3.6. Immunohistochemical Staining of TGf-*β*1, Smad2, and Smad4 in Testis

**Figure 6:**
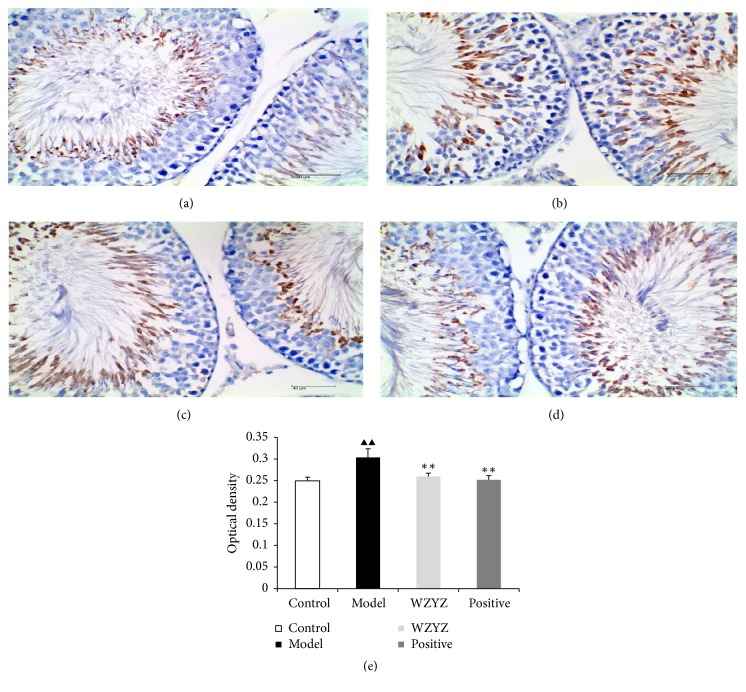
IHC analysis of TGf-*β*1 expression in testis. (a) Control group; (b) model group; (c) WZYZ group; (d) positive group; (e) quantification of TGf-*β*1 IHC staining. Bars represent means± SD of 6 rats per group. ^▲▲^p<0.01 versus the control group; ^*∗∗*^p <0.01 versus the model group.

**Figure 7:**
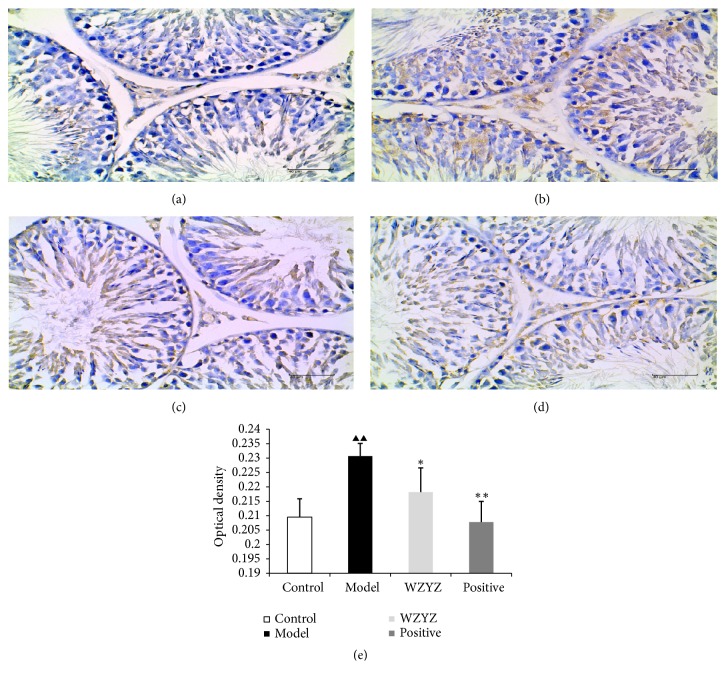
IHC analysis of Samd2 expression in testis. (a) Control group; (b) model group; (c) WZYZ group; (d) positive group; (e) quantification of Smad2 IHC staining. Bars represent means± SD of 6 rats per group. ^▲▲^p<0.01 versus the control group, ^*∗*^p <0.05 versus the model group, and ^*∗∗*^p <0.01 versus the model group.

**Figure 8:**
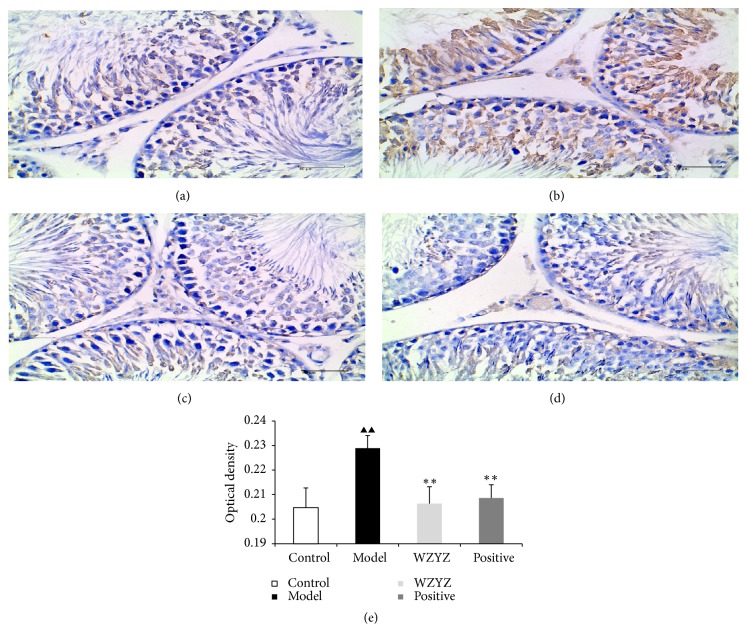
IHC analysis of Smad4 expression in testis. (a) Control group; (b) model group; (c) WZYZ group; (d) positive group; (e) quantification of Smad4 IHC staining. Bars represent means± SD of 6 rats per group. ^▲▲^p<0.01 versus the control group; ^*∗∗*^p <0.01 versus the model group.

IHC analysis was performed to ascertain the effect on TGf-*β*1 (Figure 6), Smad2 (Figure 7), and Smad4 (Figure 8) expression on the testis of the rats with oligoasthenozoospermia. TGf-*β*1 was mainly expressed in the cytoplasm of elongated spermatids, Smad2 was localized in the cytoplasm of spermatogenic, and Smad4 was primarily localized in the cytoplasm of spermatogonial and primary spermatogenic cells in the testis. The immunostaining intensity was significantly stronger in the model group compared to the control and decreased after WZYZ treatment (p<0.01 for all).

## 4. Discussion

In recent years, the incidence of male infertility has been observed to be high but the cause of male infertility is complicated. Studies have shown that oligoasthenozoospermia is a major etiological factor of male infertility and characterized by spermatogenic disorder. It can be simulated in animal models via chemical, physical, and surgical means. In general, chemical induction of oligoasthenozoospermia is convenient and has shown high specificity [26]. Tripterygium glycosides have anti-inflammatory and immunological functions, and long-term oral administration can reduce sperm counts and quality, leading to spermatogenic disorder and infertility. At present, the dosage and time for induction of oligoasthenozoospermia model by tripterygium glycosides tablet have been reported in detail. The changes in sperm morphology, kinetics, and genesis in the GTW-modelled rats are consistent with the clinical manifestations of infertility, which provide an ideal modelling method for the researchers [27].

The SD rats used in this experiment were orally administered with GTW 20mg^−1^kg once daily for four weeks. The results of previous study showed markedly lower sperm counts and motility in the model group compared to the healthy animals. At the histological level, the results of the present study showed that the modelled rats had significantly damaged spermatogenic cells and lower pathological scores in the model group, which was markedly different from that in the control group. The changes were basically consistent with the precious reports, which proved that the model was successful [28, 29]. Histopathological results showed that WZYZ formula significantly improved the testicular tissues damage.

Spermatogenesis is one of the important functions of testis. Spermatogenesis and spermatozoa maturation are dependent on the process of continuous growth and differentiation, which depends on the complete function of hypothalamic-pituitary-gonad (HPG) axis. The GnRH secreted by the hypothalamus, FSH and LH secreted by the pituitary gland, testosterone, and E2 secreted by the testis form a closed feedback loop which ensures normal spermatogenesis [30–32]. GnRH stimulates the pituitary to release FSH and LH, and the latter acts on Leydig cells to stimulate the synthesis and secretion of testosterone [33]. FSH enhances the effects of LH, and acts on the Sertoli cells to promote the development of secondary spermatocytes into spermatozoa, as well as the synthesis of androgen binding proteins which maintain high levels of testosterone in the spermatogenic microtubules. The latter is one of the key factors necessary for maintaining spermatogenesis, and testosterone deficiency affects spermatogenesis, and the vitality and fertilization ability of the spermatozoa [34–36]. Recent studies have shown that estrogen is almost involved in the whole process of spermatogenesis and is an essential hormone for spermatogenesis and maturation [37–40].

Regulation of the HPG is the key to normal reproductive function, and any dysregulation results in abnormal levels of reproductive hormones, which affect spermatogenesis. Experimental and clinical studies show fluctuating levels of reproductive hormones in both oligoasthenozoospermia patients and animal models, depending on the site of testicular damage [41–43]. Therefore, serum levels of reproductive hormones are a reliable indicator of the HGP axis function. The oligoasthenozoospermic rats showed significantly higher levels of FSH and lower levels of LH, T, GnRH, and E2 which corresponds to abnormal spermatogenesis. WZYZ restored the hormonal levels, further underscoring its therapeutic effects on the spermatogenic function in testis.

In addition to the regulation of reproductive hormones, spermatogenesis is also regulated by the cytokines secreted by various testicular cells. Transforming growth factor (TGF-*β*1), a peptide growth factor of the TGF-*β* superfamily, has a wide range of biological effects, especially regulates fertilization, embryonic development, and tissue/organ [44–46]. It regulates testicular function to maintaining testicular homeostasis and spermatogenesis [47]. In addition, related studies have shown that TGF-*β*1 is expressed in different cells of testis at different developmental stages, including spermatogenic cells, Leydig cells, and Sertoli cells, and the expression has obvious spatiotemporal characteristics [48]. Meanwhile the expression of TGF*β*1 in mammalian testis is species-specific [49]. The Smads proteins are intracellular signal transduction molecules of TGF-*β*1 that bind to other transcription factors and regulate TGF-*β*1 target gene expression. Smads proteins play an important role in the signal transduction of TGF-*β*1. According to the results of relevant research, Smads may directly or indirectly regulate sperm production and maturation, along with testicular function [50].

TGF-*β*1, Smad2, and Smad4 are expressed in the testis, and the TGF-*β*1/Smads signaling pathway is closely associated with testicular development and spermatogenesis [51, 52]. Specific targeting of this pathway improved testicular morphology and sperm production and quality in animal models [53–57]. We found that TGF-*β*1 was expressed in the cytoplasm of elongated spermatids, Smad2 was localized in the cytoplasm of spermatogenic, and Smad4 expression was shown in the cytoplasm of spermatogonial and primary spermatogenic cells. Different localization of Smad4 was reported in a previous study, likely due to the different stages of testicular development [50]. The TGF-*β*1, Smad2, and Smad4 protein expression detected by WB and mRNA levels detected by qRT-PCR were significantly increased in the model group and downregulated after WZYZ treatment.

These results showed that TGF-*β*1/Smads signal pathway is a target of the WZYZ formula to regulate spermatogenic function in the testis, providing a molecular understanding of the biological activity that prevents oligoasthenozoospermia. In addition, TGF-*β*1/Smads signal pathway might be the important target for Chinese medicine to treat oligoasthenozoospermia caused by a deficiency of kidney essence.

Due to practical constraints, the present study has some limitations. Further dissection of the WZYZ formula to treat oligoasthenozoospermia caused by deficiency of kidney essence should be further explored. Additionally, it should be examined whether other signal pathways are involved in the spermatogenic function in testis.

## 5. Conclusion

The results in the present study showed that a rat model of oligoasthenozoospermia caused by deficiency of kidney essence caused a series of disorders. We identified disorders of related reproductive hormones, histopathological changes in testis tissue, and abnormalities in the TGF-*β*1/Smads signal pathway. These disorders may induce oligoasthenozoospermia under certain conditions. The WZYZ formula treatment alleviated the symptoms of oligoasthenozoospermia caused by deficiency of kidney essence. Meanwhile, our study indicated that WZYZ exerts a role on the treatment of oligoasthenozoospermia in the rat model by restoring reproductive hormones levels and HPG axis function, along with targeting the TGF-*β*1/Smads pathway in the testis.

## Figures and Tables

**Table 1:** The different groups and treatments used.

Group	Number of animals	Time	Treatment
Control group	8	28d	Distilled water
Model group	8	28d	Tripterygium glycosides (20 mg/kg)+ Distilled water
Positive group	8	28d	Tripterygium glycosides (20 mg/kg)+ Methyltestosterone (25 mg/kg)
WZYZ formula group	8	28d	Tripterygium glycosides (20 mg/kg)+WZYZ (1.62g/kg)

**Table 2:** Primer sequence of genes.

Gene symbol	Primers	Amplicon (bp)
*β*-actin	F: GAAGATCAAGATCATTGCTCCT	118
	R: TACTCCTGCTTGCTGATCCA	
Smad2	F: ATGGTCGTCTTCAGGTGTCTCATCGG	257
	R: GGTATAGTCATCCAGAGGCGGACGTT	
Smad4	F: CGCCGTCTTCGTGCAGAGTTACTACC	253
	R: GCCAGCAGCAGCAGACAGACTGATG	
TGf-*β*1	F:AATGGTGGACCGCAACAACGCAATCT	102
	R:TCTGGCACTGCTTCCCGAATGTCTGA	

## Data Availability

The data used to support the findings of this study are included within the article.
